# Arsenite oxidation regulator AioR regulates bacterial chemotaxis towards arsenite in *Agrobacterium tumefaciens* GW4

**DOI:** 10.1038/srep43252

**Published:** 2017-03-03

**Authors:** Kaixiang Shi, Xia Fan, Zixu Qiao, Yushan Han, Timothy R. McDermott, Qian Wang, Gejiao Wang

**Affiliations:** 1 State Key Laboratory of Agricultural Microbiology, College of Life Science and Technology, Huazhong Agricultural University, Wuhan 430070, China; 2 Department of Land Resources and Environmental Sciences, Montana State University, Bozeman, MT 59717, USA

## Abstract

Some arsenite [As(III)]-oxidizing bacteria exhibit positive chemotaxis towards As(III), however, the related As(III) chemoreceptor and regulatory mechanism remain unknown. The As(III)-oxidizing bacterium *Agrobacterium tumefaciens* GW4 displays positive chemotaxis towards 0.5–2 mM As(III). Genomic analyses revealed a putative chemoreceptor-encoding gene, *mcp*, located in the arsenic gene island and having a predicted promoter binding site for the As(III) oxidation regulator AioR. Expression of *mcp* and other chemotaxis related genes (*cheA, cheY2* and *fliG*) was inducible by As(III), but not in the *aioR* mutant. Using capillary assays and intrinsic tryptophan fluorescence spectra analysis, Mcp was confirmed to be responsible for chemotaxis towards As(III) and to bind As(III) (but not As(V) nor phosphate) as part of the sensing mechanism. A bacterial one-hybrid system technique and electrophoretic mobility shift assays showed that AioR interacts with the *mcp* regulatory region *in vivo* and *in vitro*, and the precise AioR binding site was confirmed using DNase I foot-printing. Taken together, these results indicate that this Mcp is responsible for the chemotactic response towards As(III) and is regulated by AioR. Additionally, disrupting the *mcp* gene affected bacterial As(III) oxidation and growth, inferring that Mcp may exert some sort of functional connection between As(III) oxidation and As(III) chemotaxis.

Arsenic (As) is widespread in the environment and can exist in several different oxidation states and species^1^. The most common arsenic compounds relevant to human health are arsenite [As(III)] and arsenate [As(V)]^2^. Arsenic in the environment typically results from either natural geologic sources or anthropogenic activities (e.g., arsenical pesticides, fossil fuels combustion, mining activities). Arsenic contaminated drinking water has become a big enveronmental problem in many regions of the world, including Bangladesh, India, China and US^2^,^3^,^4^.

Microorganisms contribute to As biogeochemical cycling, catalyzing As(III) oxidation, As(V) reduction and As(III) methylation, which are the prominent reactions influencing As speciation and in turn strongly affect As mobility and toxicity^5^,^6^,^7^. In most As(III)-oxidizing bacteria, the expression of As(III) oxidase genes *aioBA* is regulated by a three-component signal transduction system including the periplasmic As(III)-binding protein AioX, the sensor kinase AioS and its cognate response regulator AioR^8^,^9^. In addition, the consensus DNA binding sequence GTTN(10) AAC has been documented for AioR^10^,^11^. In some cases, microbial As(III) oxidation is not only a strategy for detoxification but also a metabolic reaction to generate energy^12^,^13^. The chemoautotrophic As(III) oxidizer NT-26, and the heterotrophic As(III) oxidizers *Hydrogenophaga* sp. str. NT-14 and *Agrobacterium tumefaciens* GW4 have all been shown to obtain energy from As(III) oxidation^14^,^15^.

Microorganisms have evolved numerous abilities to respond and adapt to nutrient scarcity and environmental toxins^16^. Chemotaxis enables microorganisms to migrate towards attractants or away from repellents^17^. Generally, the regulation of bacterial chemotaxis involves a receptor referred to as the methyl-accepting chemotaxis protein Mcp, a histidine kinase CheA, and response regulator CheY^18^. A number of Mcp proteins have been characterized for sensing different ligands, and as such are critical elements of the different chemotaxis responses. Mcp proteins sense molecules that elicit the chemotaxis response by targeting specific ligands or signal molecules and control auto-phosphorylation of CheA, which then catalyzes the transfer of the phosphoryl group to a conserved aspartate of CheY^19^. The resulting CheY-Pi interacts with a flagellar motor mechanism, which causes a change in bacterial behavior, such as direction or speed of flagella rotation^19^.

Two As(III)-oxidizing bacteria, *Herminiimonas arsenicoxydans* ULPAs1 and *Rhizobium* sp. NT-26, have been shown to exhibit chemotaxis towards As(III)^10^,^20^. The average swimming speed of *H. arsenicoxydans* ULPAs1 increases 2-fold in the presence of 2 mM As(III)^20^. The flagellin FliC, hook-associated protein FliD, flagellar assembly protein FliH and flagellar hook protein FlgE are all up-regulated in response to As(III) exposure^21^,^22^. In *Rhizobium* sp. NT-26, microarray and proteomics data showed that the expression of the flagellum M-ring protein FliF and flagellin protein FlaA was induced by the presence of As(III). However, the AioR binding site was not predicted in the regulatory region of chemoreceptor genes in *Rhizobium* sp. NT-26^10^. Moreover, the specific chemoreceptor for As(III) and the regulation mechanism(s) of chemotaxis towards As(III) remain unclear.

Previously, we have shown that the growth of *A. tumefaciens* GW4 was enhanced by As(III) oxidation^15^. Furthermore, FliC levels were enhanced after As(III) exposure, and thus we proposed that strain GW4 may have a similar As(III) chemotaxis behavior to that documented for *H. arsenicoxydans* ULPAs1^20^ and *Rhizobium* sp. NT-26^10^. In this study, we show that strain GW4 indeed exhibits positive chemotaxis towards As(III). We found that there are 20 candidate *mcp* genes in the *A. tumefaciens* GW4 genome, with one of them located adjacent to *aio* operon. Further, an AioR binding site upstream of *mcp* is shown in different experiments to regulate bacterial chemotaxis towards As(III), demonstrating a regulatory linkage spanning from As(III) signal reception by a Mcp type receptor to the regulation of As(III) chemotaxis and As(III)-oxidation.

## Results

### Genetic organization of *mcp* and the prediction of AioR putative binding sites

**Figure 1:**
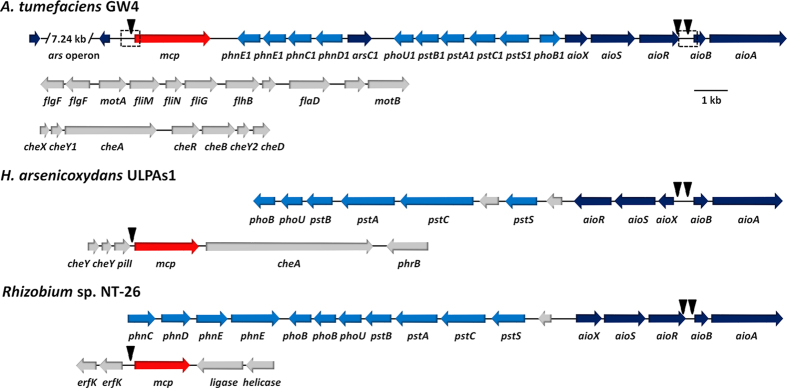
m*cp* gene in genome sequences of *A. tumefaciens* GW4 (AWGV00000000), *H. arsenicoxydans* ULPAs1 (CU207211) and *Rhizobium* sp. NT-26 (FO082820). The GI number of the *mcp* gene in GW4, ULPAs1 or NT-26 are 391324260, 500198911 and 918933804, respectively. The locations of triangles are the AioR putative binding sites. The dashed boxes approximate promoter regions that were PCR-cloned into pLSP-KT2lacZ for construction of β–galactosidase reporter fusions.

Draft genome sequencing of *Agrobacterium tumefaciens* GW4 (AWGV00000000) revealed an As(III) oxidation gene island containing the coding genes of the three-component signal transduction system (*aioXSR*) and As(III) oxidase enzyme (*aioBA*) (Fig. 1)^11^. Of the 20 candidate *mcp* genes found in the *A. tumefaciens* GW4 genome, one is located adjacent to the *aio* operon (Fig. 1). Using 12 *aioBA* regulation region sequences, including the previously reported AioR binding site for *aioBA* in the As(III)-oxidizing strains *H. arsenicoxydans* ULPAs1 and *Rhizobium* sp. NT-26^10^,^20^, a putative AioR binding motif GT[TC][AC][CG][GCT][AG][AG]A[ACT][CGA][GCT][GTA]AAC was predicted and employed to identify AioR binding motifs for both the *aioBA* and *mcp* regulatory regions in GW4 (Fig. 1)^11^. Though there are no *mcp* genes located adjacent to the arsenic island in the genomes of *H. arsenicoxydans* ULPAs1 (CU207211) and *Rhizobium* sp. NT-26 (FO082820), the AioR putative binding site was found in the regulatory regions of one of the *mcp* genes in each genome of these bacteria (Fig. 1). Thus, occurrence and position of an AioR binding site relative to a *mcp* gene and association of the *mcp* gene with adjacent *aio* genes were observations consistent with this *mcp* encoding a specific chemoreceptor that is at least somehow involved with As(III) based regulation of chemotaxis.

### Bacterial chemotaxis towards As(III) in *A. tumefaciens*

To investigate As(III) chemotaxis in GW4, the following genotypes were constructed: *aioR* deletion strain, GW4-∆*aioR*; *mcp* deletion strain GW4-∆*mcp* and their relevant complemented mutant strains, GW4-∆*aioR*-C and GW4-∆*mcp*-C. The successful mutation and complementation were confirmed by diagnostic PCR (Fig. S1) and DNA sequencing (data not shown).

**Figure 2:**
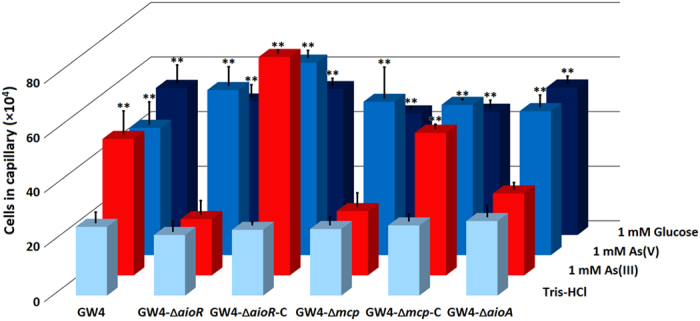
Quantification of the chemotactic response towards 1 mM As(III), 1 mM As(V) or 1 mM glucose of *A. tumefaciens* strains. Data were obtained from quantitative capillary assays. Tris-HCl (pH = 7.0) was used as a negative control. Results represent the means from at least three independent experiments and the error bars indicate standard errors. Stars represent statistical significance (**p < 0.01).

Washed As(III) induced cells were used to test the As(III) chemotactic phenotypes employing the capillary assay. The *A. tumefaciens* strains all showed a neutral response to Tris-HCl and a significant positive response to 1 mM glucose (positive control) (*p* < 0.01), indicating that the loss of *aioR* or *mcp* did not interrupt the bacterial chemotactic system for certain carbon sources (Fig. 2). Chemotaxis experiments showed positive responses towards As(III) (0.5 mM up to 2 mM, data not shown). As shown in Fig. 2, the wild-type strain GW4 showed a significant positive response to 1 mM As(III) (*p* < 0.01), but the mutant strains GW4-∆*aioR* and GW4-∆*mcp* did not (*p* > 0.5). The complemented strains GW4-∆*aioR*-C and GW4-∆*mcp*-C recovered the ability to respond to As(III) (*p* < 0.01) (Fig. 2). The results indicate that strain GW4 possesses the chemotaxis machinery needed to mount a positive As(III) chemotaxis response, and that both *aioR* and *mcp* genes are required. Also, deletion of *aioA* abolished As(III) chemotaxis (Fig. 2), indicating that As(III) chemotaxis requires As(III) oxidation. Finally, all of *A. tumefaciens* strains showed a similar increase in response to 1 mM As(V) (*p* < 0.01, Fig. 2), indicating that As(V) was also an attractant but independent of *aioA, aioR* and *mcp*, as was also observed for glucose (Fig. 2).

**Figure 3:**
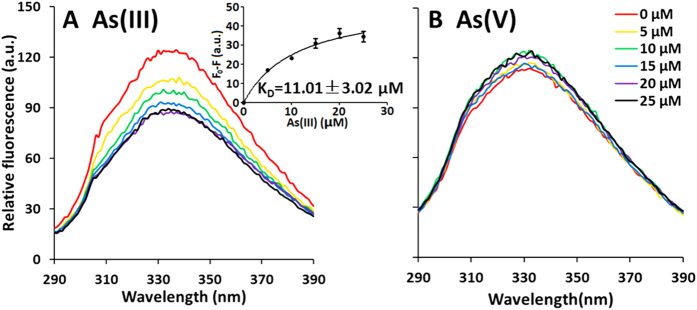
Interaction of Mcp with As(III) and As(V) as measured by relative intrinsic fluorescence. Purified Mcp (1.0 μM) was incubated with As(III) or As(V) (0, 5, 10, 15, 20 and 25 μM) for 1 h before testing. (**A**) Fluorescence quenching observed when Mcp was incubated with incremental increases of As(III). (**B**) Fluorescence quenching observed when Mcp was incubated with incremental increases of As(V). Fluorescence change at 330 nm was analyzed by GraphPad Prism 5, and K_D_ value was calculated to be 11.01 ± 3.02 μM.

To reveal how Mcp functions in *A. tumefaciens* GW4 chemotaxis towards As(III), the sensing domain of Mcp was purified (Fig. S2) and employed in tryptophan-based intrinsic fluorescence assays to examine its affinity for As(III), phosphate (Pi) or As(V) (Fig. 3). The four tryptophan residues (W_44_, W_166_, W_254_ and W_308_) in Mcp implied the protein would be a good candidate for this assay. With incremental increases of As(III), Pi or As(V), fluorescence quenching was specifically found with As(III), but not As(V) (Fig. 3) nor Pi (data not shown). The K_D_ value with As(III) was 11.01 ± 3.02 μM (Fig. 3). This data is consistent with the capillary assay results and the mutant work, implying that the Mcp protein (proximal to the *aio* operon) is an As(III) chemoreceptor, and participates in As(III) chemotaxis in *A. tumefaciens* GW4.

### As(III) chemotaxis and As(III) oxidation

**Figure 4:**
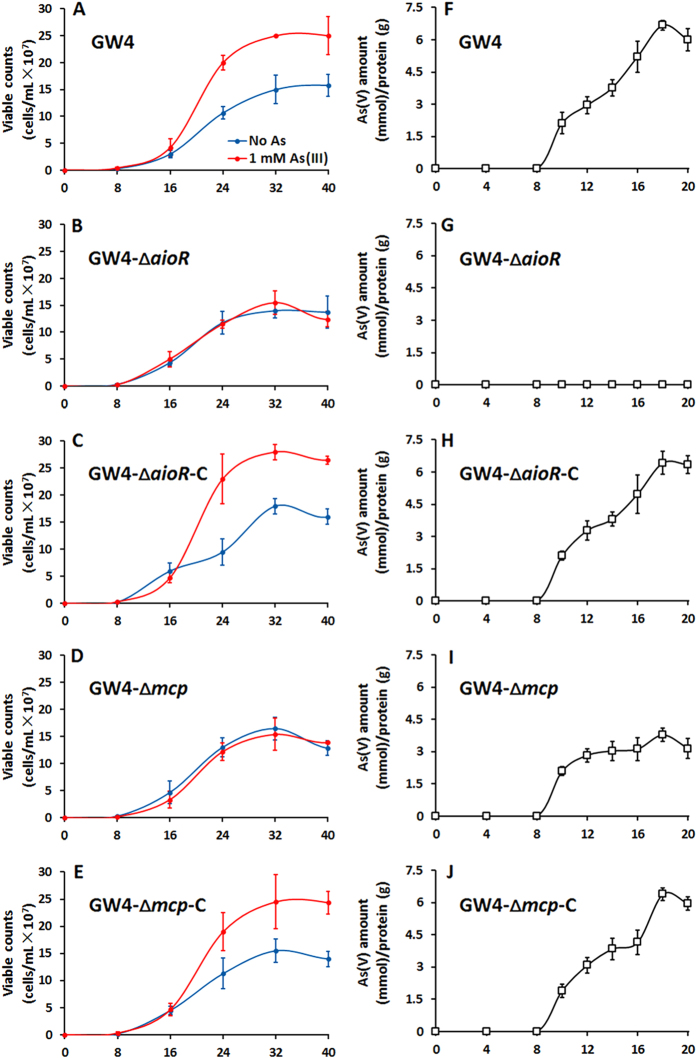
Importance of *aioR* and *mcp* on bacterial growth and As(III) oxidation efficiency in *A. tumefaciens* GW4. (**A–E**) Growth curves of strains GW4, GW4-∆*aioR*, GW4-∆*aioR*-C, GW4-∆*mcp* and GW4-∆*mcp*-C in MMNH_4_ medium with or without the addition of 1 mM As(III). (**F–J**) As(III) oxidation of the same strains. Data symbols shown in (**A**) are the same for (**B–E**). Data are shown as the mean of three replicates, with the error bars illustrating one standard deviation.

To further study the link between As(III) oxidation and As(III) chemotaxis, growth and As(III) oxidation were tested in MMNH_4_ with or without the addition of 1 mM As(III) (Fig. 4). Consistent with the previous results^15^, addition of As(III) resulted in significantly enhanced growth for GW4 (Fig. 4A), indicating As(III) oxidation in this strain yielded energy for growth^15^. Consistent with the results we previously reported with the *aioA* mutant^15^, mutant strain GW4-∆*aioR* failed to demonstrate increased growth and its As(III) oxidation phenotype was disrupted (Fig. 4B and G). As(III) oxidation efficiency in strain GW4-∆*mcp* was decreased by roughly 50% (Fig. 4I), apparently linked with lack of enhanced growth as seen in the wild type (compare Fig. 4A and D). As expected if these mutations were directly associated with the altered growth phenotype, providing *aioR* or *mcp in trans* (strain GW4-∆*aioR*-C or strain GW4-∆*mcp*-C) recovered the mutant phenotype back to wild-type GW4 status with respect to growth response to added As(III) (Fig. 4C,H,E and J). Moreover, As(III) resistance was reduced in the GW4-∆*mcp* mutant but recovered in the complemented strain (Fig. S3). Taken together, these results establish a firm linkage among As(III) chemotaxis, As(III) oxidation, As(III) resistance and growth.

### Expression of *aioA, mcp* and other chemotactic related genes

**Figure 5:**
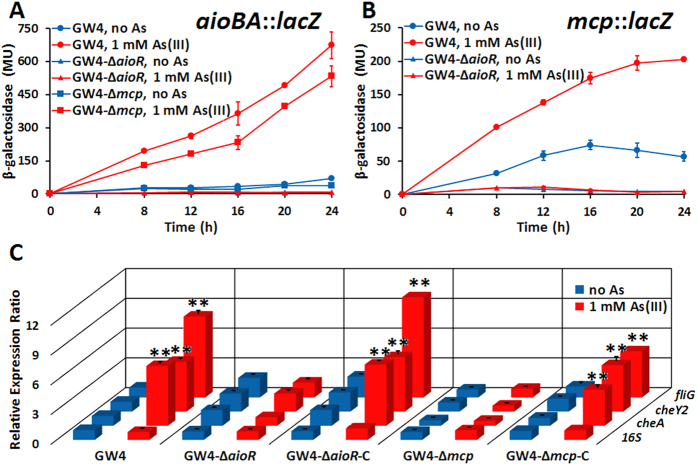
Influence of As(III) on the expression of *aioA, mcp* and chemotaxis related genes. (**A**) Expression of *aioBA*::*lacZ* in strains GW4, GW4-∆*aioR* and GW4-∆*mcp* in MMNH_4_ medium with or without the addition of 1 mM As(III). (**B**) Expression of *mcp*::*lacZ* in strains GW4, GW4-∆*aioR* and GW4-∆*mcp* in MMNH_4_ medium with or without the addition of 1 mM As(III). (**C**) Expression of *cheA, cheY2* and *fliG* genes detected by qRT-PCR in different strains in MMNH_4_ medium with or without the addition of 1 mM As(III). Data are shown as the mean of three replicates, with the error bars illustrating one standard deviation. The statistical significance is represented by stars (**p < 0.01).

To further investigate the relationship between AioR and As(III) chemotaxis, *lacZ* reporter gene assays were performed. As expected, *aioBA*::*lacZ* expression was significantly induced by As(III) in the wild-type GW4 but not in GW4-∆*aioR* (Fig. 5A), and thus consistent with the null As(III) oxidation phenotype of this mutant (Fig. 4G). Furthermore, *aioBA*::*lacZ* induction was significantly attenuated in strain GW4-∆*mcp (p* < 0.01; Fig. 5A), which is also consistent with the decreased As(III) oxidation efficiency in GW4-∆*mcp* mutant (Fig. 4I). Expression of *mcp*::*lacZ* was significantly upregulated by As(III) in the wild-type strain GW4, however it failed to induce in strain GW4-∆*aioR* (Fig. 5B). The above results indicated that AioR regulates the expression of *aioBA* and *mcp*, and disruption of *mcp* appears to influence *aioBA* expression.

Given the apparent influence of AioR on *mcp* expression, additional experiments using quantitative reverse transcriptase (qRT)-PCR were performed to assess the effect of As(III) on the expression of other chemotaxis related genes, namely *cheA, cheY2* or *fliG*. These genes were all induced by As(III) in strain GW4, but the transcription levels were significantly decreased in mutant strains GW4-∆*aioR* and GW4-∆*mcp*, and recovered in the complemented strains GW4-∆*aioR*-C and GW4-∆*mcp*-C (Fig. 5C). The results are consistent with the null As(III) chemotaxis phenotypes in strains GW4-∆*aioR* and GW4-∆*mcp* (Fig. 2), again implicating AioR and Mcp control As(III) chemotaxis in *A. tumefaciens* GW4.

### AioR regulation of As(III) chemotaxis

**Figure 6:**
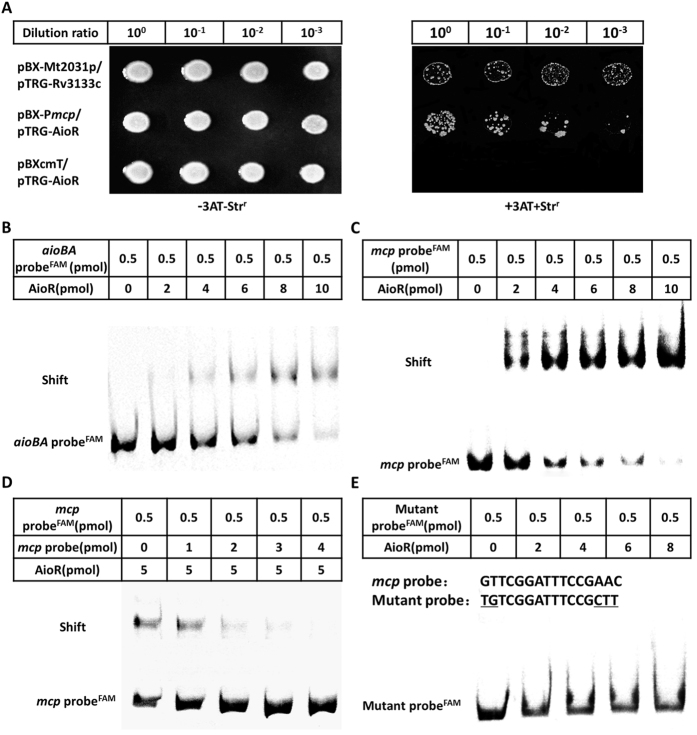
Bacterial one-hybrid and EMSA analyses for the interaction between AioR and *mcp* promoter DNA. (**A**) Bacterial one-hybrid assay. Co-transformants containing pBX-Mt2031p/pTRG-Rv3133c were employed as positive controls (CK+), while co-transformants containing pBXcmT/pTRG-AioR was used as negative controls (CK−). Cells of CK+, pBX-P*mcp*/pTRG-AioR, and CK- were grown to OD_600_ of 1.0 and 2 μL of each was spotted onto His-selective medium (+3AT, +Str^r^) and LB plate (−3AT, −Str^r^). (**B–E**) EMSA assays. FAM-labeled *aioBA* probe (**B**) and *mcp* probe (**C**) both interacted with AioR protein. (**D**) Competition experiments including labeled and unlabeled *mcp* probe. (**E**) Mutant FAM-labeled *mcp* probe (the mutation sites are underlined) interaction with AioR protein. The amounts of DNA probes and AioR were shown in the above table of each panel.

Bacterial one-hybrid assays were then performed to examine the interaction between AioR and the *mcp* promoter region^23^. The regulatory sequence of *mcp* containing a putative AioR binding site was cloned into the reporter vector pBXcmT, while the AioR coding region was introduced into the pTRG vector. Detection of protein-DNA interactions is based on transcriptional activation of the *His* reporter gene, which allows growth in the presence of 3-amino-1,2,4-triazole (3-AT), a competitive inhibitor of the His3 enzyme^23^. Positives are verified by using the gene which confers streptomycin resistance as a secondary reporter^23^. Both constructs were then transferred into reporter strain *E. coli* XL1-Blue MRF’ Kan (Stratagene). The reporter strain containing pBX-P*mcp* and pTRG-AioR displayed similar growth as the positive control (pBX-Mt2013p/pTRG-Rv3133c) on the screening plate containing 3-AT and Str, whereas the negative control could not. This indicated that AioR could interact with the *mcp* regulatory region *in vivo* (Fig. 6A).

AioR binding ability was then addressed *in vitro* using two different electrophoretic mobility shift assays (EMSA). The purified His_6_-tag DNA binding domain of AioR (Fig. S2) was incubated with a 170 bp *aioBA* regulatory sequence (contains one AioR putative binding site, as positive control) or a 259 bp *mcp* regulatory sequence. Increasing AioR in the binding assay resulted in electrophoretic shifts of FAM-labeled *aioBA* or *mcp* DNA substrates (Fig. 6B). Ethidium bromide staining of unlabeled DNA probes also illustrated shifts (Fig. 6C). The unlabeled *mcp* DNA substrate could competitively inhibit AioR binding with the labeled *mcp* DNA substrate (Fig. 6D). In addition, AioR was incapable of binding with the mutated *mcp* regulatory sequence that lacks the conserved, putative binding motif (Fig. 6E). These results again indicated that AioR could interact with the *mcp* regulatory region *in vitro*.

**Figure 7:**
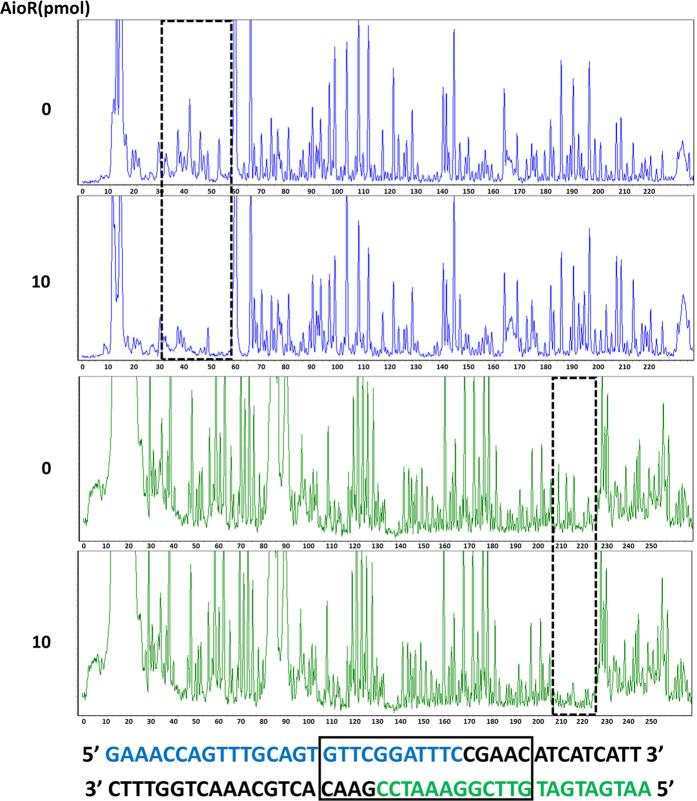
DNase I foot-printing assays for the *mcp* loci. Electropherogram-based visualization for protection pattern of *mcp* promoter after digestion with DNase I following incubation with (in the bottom, 10 pmol) or without (on the top, 0 pmol) AioR protein. The fluorescence signal of the labeled DNA fragments (FAM-labeled sense strand and HEX-labeled antisense strand) is plotted against the sequence length of the fragment. The DNase I foot-printing analysis data of both strands are showed below and the protected region is framed.

To further confirm AioR binding and to precisely determine the AioR binding site, DNase I foot-printing assays were conducted with the fluorescence (5′-FAM or 5′-HEX) labeled *mcp* 259 bp regulatory region. The results from electropherograms of both strands with different AioR concentrations showed that the GTTCGGATTTCCGAAC region upstream of *mcp* was obviously protected from DNase I digestion (Fig. 7), which is consistent with the reported AioR binding site^11^. Thus, these experiments demonstrated AioR can bind the predicted sequence in the regulatory region of *mcp*, regulate the expression of *mcp*, and is involved in the regulation of chemotaxis towards As(III) in *A. tumefaciens* GW4.

## Discussion

To date, As(III) chemotaxis has been reported in two As(III)-oxidizing strains, *H. arsenicoxydans* ULPAs1 and *Rhizobium* sp. NT-26^10^,^20^, however the As(III) chemoreceptor and the molecular mechanism(s) have not yet been demonstrated. The general aim of this study was to expand upon our understanding of the As(III) chemotactic response using the As(III)-oxidizing strain *A. tumefaciens* GW4. Herein, we first provide evidence from a variety of different approaches and experiments that are internally consistent with respect to explaining how and why strain GW4 exhibits positive motility towards As(III).

Of the 20 open reading frames annotated as *mcp* in the GW4 genome, we focused on the putative *mcp* located adjacent to the *aio* operon, assuming its location would be most promising with respect to potential involvement with As(III) chemotaxis as well as there being a putative AioR binding site in its promoter region (Fig. 1). This assumption proved to be reasonable because this gene: (1) is essential to As(III) chemotaxis (Fig. 2); (2) encodes a protein that binds As(III) (Fig. 3); (3) encodes a protein somehow involved with translating As(III) oxidation efficiency for enhancing growth (Fig. 4D); (4) is regulated by the As(III) regulator AioR (Fig. 5); and (5) exhibits binding interaction with AioR (Figs 6 and 7). These properties are completely consistent with one another and highly relevant to As(III) chemotaxis. In particular, regulatory control by AioR (Fig. 5) directly implicates that this *mcp* is part of the As(III) response that links As(III) sensing, positive As(III) chemotaxis and As(III) stimulated-growth (Fig. 4; Fig. S3). To our knowledge, this is the first experimental evidence specifically identifying an As(III) chemoreceptor distinct from AioX^9^ or ArsR^24^, and that is linked to AioR in a regulatory fashion. In the case of chemotaxis, however, the Mcp As(III) receptor levels in the cell are ultimately controlled by AioR, and so in a regulatory context Mcp As(III) sensing is a downstream activity rather than the initial detection component that initiates the As(III) response. Evidence of this Mcp acting as an As(III) chemotaxis receptor derives from experiments showing loss of As(III) chemotaxis in the Δ*mcp* mutant (Fig. 2) and shifts in Mcp tryptophan fluorescence inferring As(III) binding, but not As(V) nor Pi (Fig. 3). These ligand binding properties are similar to AioX^9^. A positive As(V) chemotaxis response in all genotypes tested (Fig. 2) implies a different system is involved for sensing As(V). Other key proteins involved in a chemotaxis response are the CheAY signal transduction components as well as the synthesis of the flagellum proteins^16^. Importantly, regulatory control of these elements also involved AioR, Mcp and As(III) (Fig. 5), providing yet additional evidence of a coordinated As(III) oxidation and chemotaxis response.

Being a toxic metalloid element, As(III) is reasonably considered as a repellent to most microorganisms^25^,^26^. However, its role as an attractant to As(III)-oxidizing bacteria such as *H. arsenicoxydans* ULPAs1^20^, *Rhizobium* sp. NT-26^10^ and now *A. tumefaciens* GW4^15^ (Fig. 2) is likely not coincidental because all of these microorganisms sharing a common trait of being able to use As(III) as an energy source^12^,^15^,^20^. The link between As(III) oxidation, energy generation, and chemotaxis is clear; i.e., disruption of *aioR, aioA*^15^ or *mcp* eliminates As(III) oxidation and the accompanying enhanced growth (Fig. 4), and negatively impacts As(III) chemotaxis (Fig. 2). Similar results were reported for *H. arsenicoxydans* ULPAs1 and *Rhizobium* sp. NT-26^10^,^20^. It is reasonable to conclude that As(III) oxidation generates energy for the chemotaxis response to As(III) and for flagellum rotation^10^, and thus a functional connection between As(III) oxidation and As(III) chemotaxis is not necessarily surprising.

One of the more novel observations derived from these studies concerns the apparent functional relationship between Mcp, As(III) oxidation and growth. The As(III) oxidation efficiency of the GW4-Δ*mcp* was significantly attenuated, compared with the wild type (Figs 4F vs 4I). Furthermore, the *aioBA* induction profile in GW4-Δ*mcp* was remarkably perturbed (Fig. 5A) and indeed As(III)-enhanced growth was absent (Fig. 4D). All phenotypes were restored to wild type in the complemented strain GW4-Δ*mcp*-C (Fig. 4E). Taken together, all these observations indicate a clear functional connection between As(III) oxidation, As(III) chemotaxis and the As(III) stimulated-growth. Mcp involvement in As(III) stimulated-growth is reasonably tied to the acquisition of As(III) and the energy produced by As(III) oxidation. Impairing As(III) oxidation by roughly 50% resulted in loss of As(III) enhanced growth. We also note here that strain GW4 also exhibits positive chemotaxis towards Sb(III) (data not shown), indicating a functional relationship between Mcp and Sb(III) oxidation, or the inability of Mcp to differentiate between As(III) and Sb(III).

While bacterial chemotaxis *per se* is indeed a well-documented and characterized concept, it has always been studied in the context of compounds expected to be either of nutritional value (positive chemotaxis) or a potential toxin (negative chemotaxis). This study breaks from this tradition by illustrating positive chemotaxis to a known toxic molecule, As(III). Our study also provides a clear logical molecular blueprint for why and how this occurs. Specifically, positive chemotaxis to As(III) is governed by the same regulatory circuitry that controls the expression of genes essential for As(III) oxidation, a process that generates energy for this bacterium. So, our data documents how some microorganisms deviate from the norms established in textbooks.

At a second level, even though As(III) chemotaxis has been reported in the As(III)-oxidizing strains, *H. arsenicoxydans* ULPAs1 and *Rhizobium* sp. NT-26, a regulatory mechanism was lacking. The current study now provides a very clear understanding of the As(III) sensing mechanism involved, and strengthens the literatures by illustrating a function for one of the many different open reading frames that are annotated as *mcp* in bacterial genomes but remain uncharacterized with respect to encoded function. The specific As(III) sensing and binding Mcp protein is now known (we identified three *mcp* genes in strains ULPAs1, NT-26 and GW4, see Fig. 1). The involvement of AioR is logical. This protein is well known to regulate As(III) oxidation via positive control of the As(III) oxidase genes, but is now shown that its regulatory profile includes As(III) chemotaxis. Of particular novelty is the unexpected observation that this Mcp exerts some type of positive and essential feedback regulatory activity on As(III) oxidation.

### Summary

This study extends our knowledge of how As(III) chemotaxis is part of the bacterial physiological response to As(III). The *mcp* gene adjacent to the *aio* operon is essential for this process, it is positively regulated by AioR and encodes a protein that binds As(III) that in turn initiates the As(III) chemotaxis response. This enables the cells to swim towards As(III), which is then used as an energy source for enhanced growth. Deletion of *mcp* attenuated As(III) oxidation efficiency and eliminated As(III)-enhanced growth.

## Methods

### Strain and culture condition

Bacterial strains and plasmids used are listed in Table S1. *A. tumefaciens* GW4 was grown at 28 °C in a defined minimal mannitol medium MMNH_4_^27^ containing 55 mM mannitol as the primary carbon source and modified to contain 0.1 mM phosphate. As noted, 1.0 mM NaAsO_2_ [As(III)] was added to the medium. *Escherichia coli* strains were grown in lysogeny broth (LB) (peptone, 10 g L^−1^; yeast extract, 5 g L^−1^; NaCl, 10 g L^−1^) at 37 °C. When required, 50 μg mL^−1^ of kanamycin (Kan), 50 μg mL^−1^ of gentamicin (Gen), 5 μg mL^−1^ of tetracycline (Tet), 50 μg mL^−1^ chloramphenicol of (Cm) or 100 μg mL^−1^ of ampicillin (Amp) was added.

### Prediction of AioR putative binding sites

The putative AioR binding motif was predicted by MEME online software (http://meme-suite.org/tools/meme)^11^. Using the putative AioR binding motif, the AioR putative binding sites in the genomes of *A. tumefaciens* GW4 (AWGV00000000), *H. arsenicoxydans* ULPAs1 (CU207211) and *Rhizobium* sp. NT-26 (FO082820) were predicted by FIMO online software (http://meme-suite.org/tools/fimo), with the parameter of match value of 0.0001.

### Construction of *aioR* or *mcp* mutants and complementation

In-frame deletions in *aioR* and *mcp* were constructed using cross-over PCR as described by Link and colleagues^28^. Primers used for deletion constructions are listed in Table S2. The final constructs in plasmids pJQ-*aioR* and pJQ-*mcp* were mobilized into GW4 via conjugation with *E. coli* strain S17-1. Single cross-over mutants were identified on MMNH_4_-Gen agar, which were then screened on MMNH_4_ agar containing 25% sucrose, selecting for sucrose resistance resulting from resolution of the merodiploid and resulting in the double cross-over. Gen^Sen^, sucrose-resistant isolates were screened by PCR to verify the GW4-Δ*aioR* mutant or GW4-Δ*mcp* mutant. For complementation, the *aioR* was PCR-cloned as a *Sal*I-*Hin*dIII fragment (primers AioR-C-F and AioR-C-R, Table S2) into pCT-Zori, yielding pCT-Zori-*aioR*. The *mcp* was PCR-cloned as a *Sal*I-*Xba*I fragment (primers Mcp-C-F and Mcp-C-R, Table S2) into pCT-Zori, resulting in pCT-Zori-*mcp*. These plasmids were transformed into *E. coli* S17-1 and conjugated into the GW4-∆*aioR* or GW4-∆*mcp*, yielding the complemented strains GW4-Δ*aioR*-C or GW4-∆*mcp*-C. Deletions and complementations were confirmed by PCR using primers AioR-up-F/AioR-dn-R, AioR-in-F/AioR-in-R, Mcp-up-F/Mcp-dn-R and Mcp-in-F/Mcp-in-R (Table S2), along with sequence confirmation.

### Chemotaxis assay

Chemotaxis was tested by capillary assays^29^. Overnight cultures (OD_600_ = 0.7–0.8) were each inoculated (10 μL) into 5 mL of MMNH_4_ with or without the addition of 1 mM of As(III) and incubated at 28 °C for 16 h with 100 rpm shaking. Cells were then harvested by centrifugation (3,000 rpm for 5 min) and resuspended in 5 mL 50 mM Tris-HCl (pH = 7.0). The capillary tubes were filled with 10 μL chemo-effector solution [Tris-HCl (pH = 7.0) containing 0.5–10 mM As(III), 1 mM As(V) or 1 mM glucose], and then the open ends of capillary tubes were blocked by 1% agarose gel. The capillary tubes were then vertically placed in the bacterial suspension. After 10 min incubation at room temperature, the number of cells in the capillary was quantified by colony-forming units on LB agar.

### Plasmid construction, expression and purification of AioR and Mcp

Expression of the entire AioR proved difficult, and so the Protein Blast online program was used to predict its DNA binding domain (HTH). The *aioR* (from 868 bp to 1,329 bp) was PCR-cloned as a *Eco*RI-*Not*I fragment using primers AioR-HTH-F and AioR-HTH-R (Table S2) into pET-28a(+), resulting in pET-28a-*aioR*. Cells containing pET-28a-*aioR* were induced at OD_600_ of 0.3 by adding 0.02 mM IPTG, and cultivated at 20 °C for 12 h.

Online software HMMTOP (http://www.enzim.hu/hmmtop/html/submit.html) and TMPRED (http://www.ch.embnet.org/software/TMPRED_form.html) were used to predict the Mcp extracellular membrane sensing domain (from 43 aa to 325 aa), which was PCR-cloned as a *Eco*RI-*Hin*dIII fragment using primers Mcp-As-F and Mcp-As-R (Table S2) into pET-28a(+), resulting in pET-28a-*mcp*. Cells containing pET-28a-*mcp* were induced at OD_600_ of 0.3 by adding 0.3 mM IPTG and cultivated at 28 °C for 4 h.

Cells containing pET-28a-*aioR* were cultivated at 20 °C for 12 h and cells containing pET-28a-*mcp* were cultivated at 28 °C for 4 h. They were then harvested by centrifugation (7,000 g for 10 min at 4 °C) and resuspended in borate saline buffer (pH = 8.0)^30^ with 20 mM imidazole. Unbroken cells and fragments were collected by centrifugation at 7,000 rpm for 10 min. The supernatant was mixed with 2 mL of Ni-NTA His-Bind Resin (7 seabiotech) and gently agitated at 4 °C for 1 h to allow the polyhistidine-tagged protein to bind with the resin. The resin was washed with 10 mL of borate saline buffer containing 60 mM imidazole, then eluted with 5 mL borate saline buffer containing 300 mM imidazole. Fractions were collected and analyzed by SDS-PAGE, and protein concentrations determined spectrophotometrically (NanoDrop 2000, Thermo).

### Mcp-binding with As(III), As(V) and phosphate (Pi)

Purified truncated Mcp was incubated with different concentrations (0, 5, 10, 15, 20 or 25 μM) of As(III), As(V) or Pi at room temperature for 1 h. Tryptophan fluorescence was monitored between 290 and 390 nm with a fluorescence spectrophotometer (PerkinElmer, Massachusetts, USA). The K_D_ value was calculated by GraphPad Prism 5^9^.

### Culturing and As(III) oxidation test

Overnight cultures of GW4, GW4-Δ*aioR*, GW4-Δ*aioR*-C, GW4-Δ*mcp* and GW4-Δ*mcp*-C (OD_600_ = 0.7–0.8) were each inoculated (200 μL) into 100 mL of MMNH_4_ with or without 1 mM of As(III) and incubated at 28 °C for up to 48 h with 100 rpm shaking^15^. At designated times, culture samples were taken for viable plate counts or measured for total protein content by the Bradford protein assay^31^. Monitoring As(III)/As(V) concentration used high-performance liquid chromatography with hydride-generation atomic fluorescence spectroscopy (HPLC-HG-AFS) (Beijing Titan Instruments)^32^. The As(III) oxidation efficiency was calculated using the As(V) amount (mmol)/total protein (g).

### Reporter gene assay of *aioA* and *mcp*

The reporter gene assays in this study were tested by β-galactosidase activity^33^. The promoter regions of *aioBA* and *mcp* were predicted by BPROM online software (http://linux1.softberry.com/berry.phtml?topic=bprom&group=programs&subgroup=gfindb). The promoter regions of *aioBA* and *mcp* were amplified from strain GW4 (primers listed in Table S2) and introduced into the *Eco*RI-*Bam*HI sites of pLSP-KT2lacZ (Table S1). The resulting plasmids were then introduced into *A. tumefaciens* strains via conjugation as noted above. Overnight cultures of strains (OD_600_ = 0.7–0.8) were each inoculated (200 μL) into 100 mL of MMNH_4_ with or without the addition of 1 mM As(III) and incubated at 28 °C with 100 rpm shaking. During the incubation, β-galactosidase assays were conducted as described previously^33^.

### Quantitative RT-PCR analysis

Overnight cultures (OD_600_ = 0.7–0.8) were inoculated into 100 mL MMNH_4_ medium with or without the addition of 1 mM As(III) and incubated at 28 °C with 100 rpm shaking. Samples used for RNA isolation were taken after 16 h cultivation (mid log phase). Total RNA was extracted by Trizol (Invitrogen) and incubated with RNase-free DNase I (Takara) at 37 °C to remove the genomic DNA, which was then terminated by addition of 50 mM EDTA at 65 °C for 10 min. After determining the concentration of RNA by spectrophotometry (NanoDrop 2000, Thermo), 300 ng total RNA was reverse transcribed into cDNA with RevertAid First Strand cDNA Synthesis Kit (Thermo). The resulting cDNA was diluted 10-fold for real-time RT-PCR analysis using SYBR^®^ Green Realtime PCR Master Mix (Toyobo) with primers listed in Table S2. Quantitative RT-PCR was performed by ABI VIIA7 in 0.1 mL Fast Optical 96-well Reaction Plate (ABI). Each reaction was replicated three times to estimate error. Gene expression was normalized by the 2^−∆∆CT^ analysis with an iQ5 Real-Time PCR Detection System (Bio-Rad, USA)^34^. The ANOVA analysis was performed with Excel 2013.

### Bacterial one-hybrid system assay

The interaction of AioR and the *mcp* promoter DNA was tested *in vivo* using a bacterial one-hybrid system as described previously^23^. The *aioR* coding region was amplified (primers listed in Table S2) and cloned into the *Not*I-*Eco*RI sites of the pTRG vector (Stratagene), generating plasmid pTRG-AioR. The regulatory region of *mcp* was amplified (primers listed in Table S2) and inserted directly into the *Xcm*I site of pBXcmT, yielding pBX-P*mcp*. Both of recombinant plasmids were co-transformed into the reporter strain *E. coli* XL1-Blue MRF’ Kan (Stratagene). After 3–4 d of cultivation at 28 °C on a selective screening medium plate containing 20 mM 3-amino-1,2,4-triazole (3-AT), 16 μg mL^−1^ Str, 15 μg mL^−1^ Tet, 34 μg mL^−1^ Cm, and 50 μg mL^−1^ Kan, the co-transformant growth was scored. In addition, co-transformants containing pBX-Mt2031p/pTRG-Rv3133c were employed as positive controls^23^, while co-transformants containing pBXcmT/pTRG-AioR were used as negative controls.

### EMSA

To identify the putative AioR binding site in the *mcp* regulatory region, a 259 bp fragment containing the putative AioR binding site of the *mcp* regulatory region was amplified using Mcp-box-F/Mcp-box-R (Table S2). The primer Mcp-box-F was labeled with fluorophore 6-carboxy-fluorescein (FAM) when needed. EMSA was carried out with a 0.5 pmol labeled probe and increasing concentrations of AioR (from 0 to 10 pmol). A 170 bp fragment of the *aioBA* regulatory region was amplified using AioBA-box-F/AioBA-box-R (Table S2) as positive control. For competition assays, 1, 2, 3 and 4 pmol unlabeled probes were added to reaction mixtures containing 5 pmol AioR and the 0.5 pmol labeled probe. The mutant probe was produced by Fast Mutagenesis System (TransGen Biotech). All reaction mixtures were incubated at 28 °C for 30 min in binding buffer [20 mM Tris-HCl, pH 7.0; 50 mM NaCl; 1 mM dithiothreitol (DTT); 10 mM MgCl_2_; 100 μg mL^−1^ bovine serum albumin (BSA)] and then loaded onto a 6% native PAGE. After 3 h of running at 80 V in 1× TGE buffer, gels were exposed to a phosphor imaging system (Fujifilm FLA-5100)^35^.

### DNase I foot-printing assay

The fluorescence-labeled probes and the reaction system were the same as in EMSA. The mixtures were treated with DNase I (0.05 unit, Fermentas) at room temperature for 10 min. Then the reaction was stopped by making the reaction mix 50 mM EDTA and incubated in a water bath at 65 °C for 10 min. The digested DNA fragments were purified with the Nucleic Spin^®^ Gel and PCR Clean-up (MACHEREY-NAGEL) and eluted with 40 μL distilled water. The purified DNA was assayed with Applied Biosystems 3730XL DNA analyzer (manufactured by Tsingke Company, Wuhan). The results were analyzed with GeneMarkerV1.65^36^.

## Additional Information

**How to cite this article:** Shi, K. *et al*. Arsenite oxidation regulator AioR regulates bacterial chemotaxis towards arsenite in *Agrobacterium tumefaciens* GW4. *Sci. Rep.* **7**, 43252; doi: 10.1038/srep43252 (2017).

**Publisher's note:** Springer Nature remains neutral with regard to jurisdictional claims in published maps and institutional affiliations.

## Supplementary Material

Supplementary Information
