# Supplementary material for: Arsenite oxidation regulator AioR regulates bacterial chemotaxis towards arsenite in Agrobacterium tumefaciens GW4
Source: Sci Rep. 2017 Mar 3;7:43252. doi: 10.1038/srep43252 (PMC5335332; doi:10.1038/srep43252)
Supplement: Supplementary Information [file srep43252-s1.pdf]

Arsenite oxidation regulator AioR regulates bacterial chemotaxis towards arsenite in *Agrobacterium tumefaciens* GW4

Kaixiang Shi[1], Xia Fan[1], Zixu Qiao[1], Yushan Han[1], Timothy R. McDermott[2], Qian Wang[1, 2]*, Gejiao Wang[1]*

[1] *State Key Laboratory of Agricultural Microbiology, College of Life Science and Technology, Huazhong Agricultural University, Wuhan 430070, China*

[2] *Department of Land Resources and Environmental Sciences, Montana State University, Bozeman, MT 59717, USA*

* Corresponding authors:

Gejiao Wang, State Key Laboratory of Agricultural Microbiology, Huazhong Agricultural University, Wuhan 430070, China. Phone: 86-27-87281261; E-mail: gejiao@mail.hzau.edu.cn

Qian Wang, Dept. Land Resources and Environmental Sciences, Montana State University, Bozeman, MT 59717, USA. Phone: 406-599-6873; E-mail: qianwang@mail.hzau.edu.cn

Running title: AioR regulates chemotaxis towards As(III)

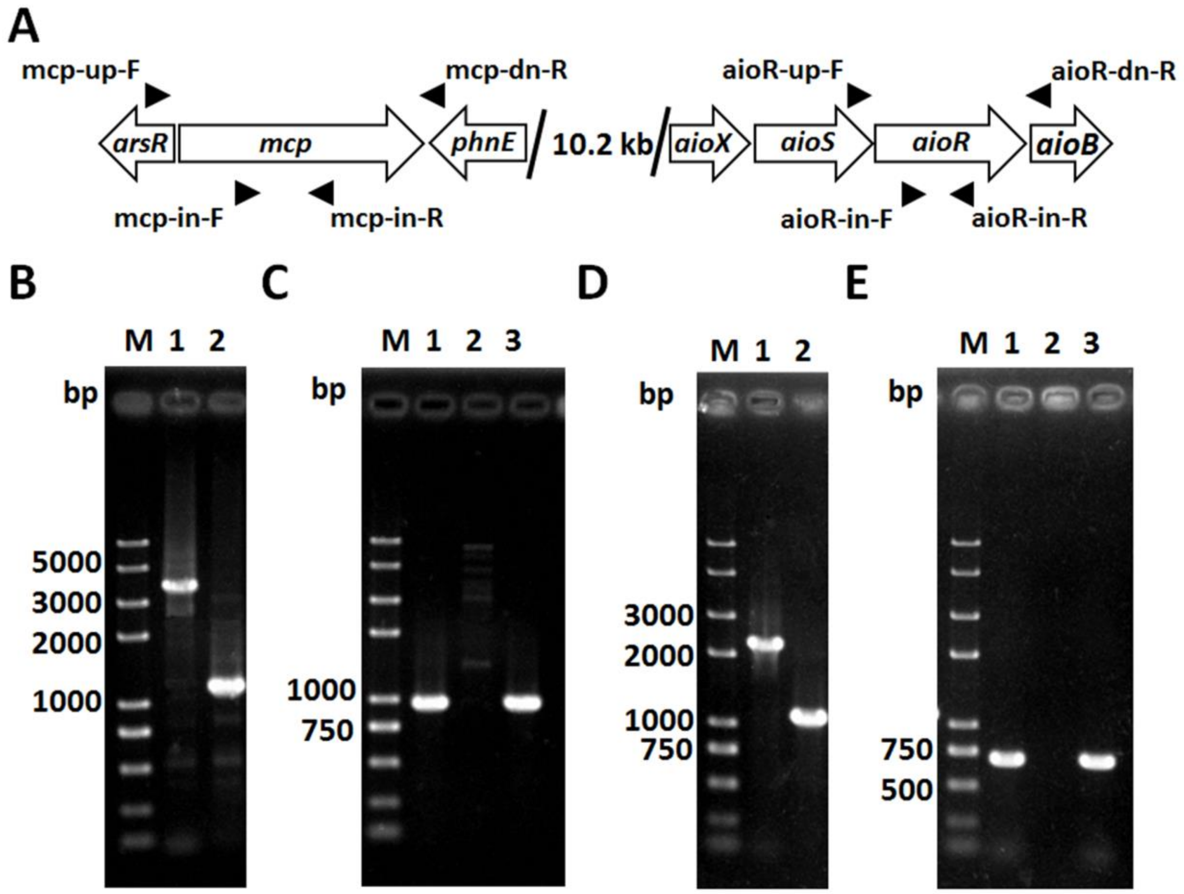

Figure S1. Full-length gels are presented in Supplementary Figure S4 (**B-C**) and Figure S5 (**D-E**). Diagnostic PCR confirming the mutant strains GW4-Δ*mcp* (**B-C**) and GW4-Δ*aioR* (**D-E**) and their complementation strain GW4-Δ*mcp*-C (**C**) and GW4-Δ*aioR*-C (**E**). (**A**) The physical map of *mcp* and *aioR* genes in *A. tumefaciens* GW4, and the primer locations used to confirm the GW4-Δ*mcp*, GW4-Δ*mcp*-C, GW4-Δ*aioR* and GW4-Δ*aioR*-C. (**B**) PCR amplicons using primers Mcp-up-F and Mcp-dn-R. (**C**) PCR amplicons using primers Mcp-in-F and Mcp-in-R. (**D**) PCR amplicons using primers AioR-up-F and AioR-dn-R. (**E**) PCR amplicons using primers AioR-in-F and AioR-in-R. For all panels: Lane 1, strain GW4, lane 2, *mcp* or *aioR* mutant strain, and lane 3, the complemented strain GW4-Δ*mcp*-C or GW4-Δ*aioR*-C. M, the molecular weight marker (DL 2000 plus). Amplicon identities were confirmed by DNA sequencing.

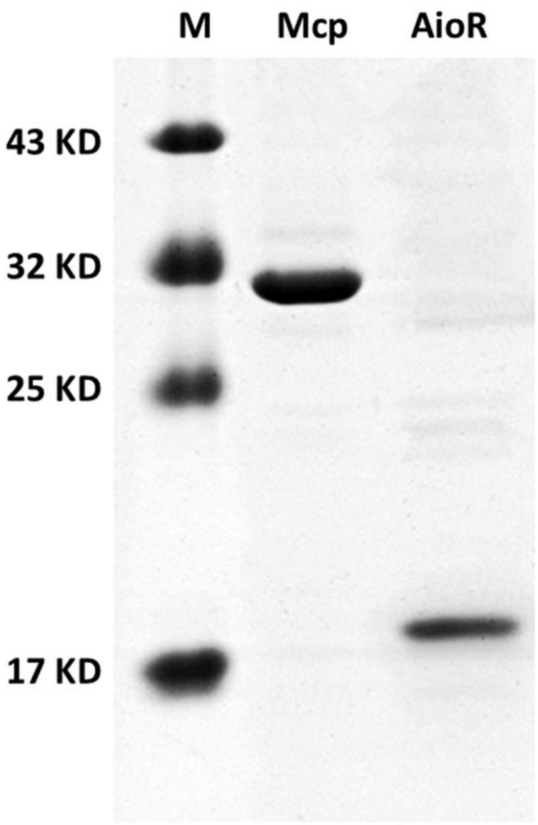

Figure S2. Over-expressed Mcp and AioR with a His_6 tag in *E. coli* BL21. The Mcp protein was purified from the 43 aa to 325 aa, which is the substrate binding domain predicted by Tmpred and CCTOP online software, without the transmembrane part. AioR protein was purified from the 318 aa to 442 aa, which is the DNA binding domain (HTH) predicted by Protein Blast online software.

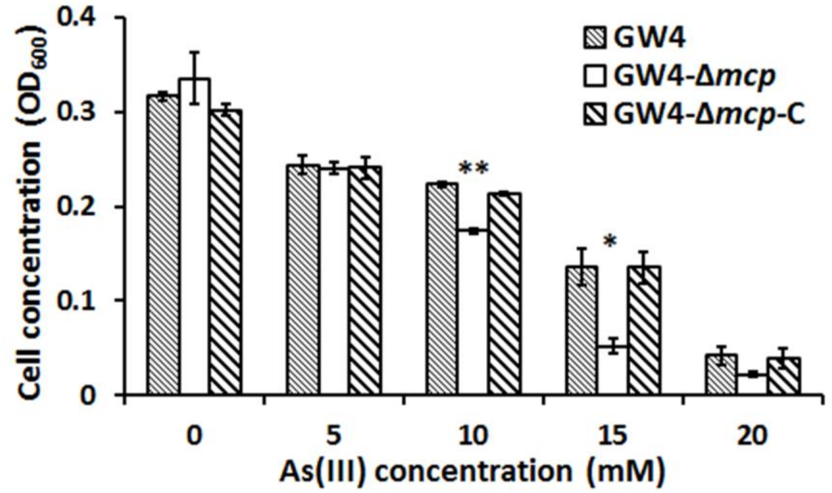

Figure S3. Effect of As(III) on bacterial growth in *A. tumefaciens* GW4. Data are shown as the mean of three replicates, with the error bars illustrating one standard deviation. The significance was represented by stars. * means $p < 0.05$, while ** means $p < 0.01$.

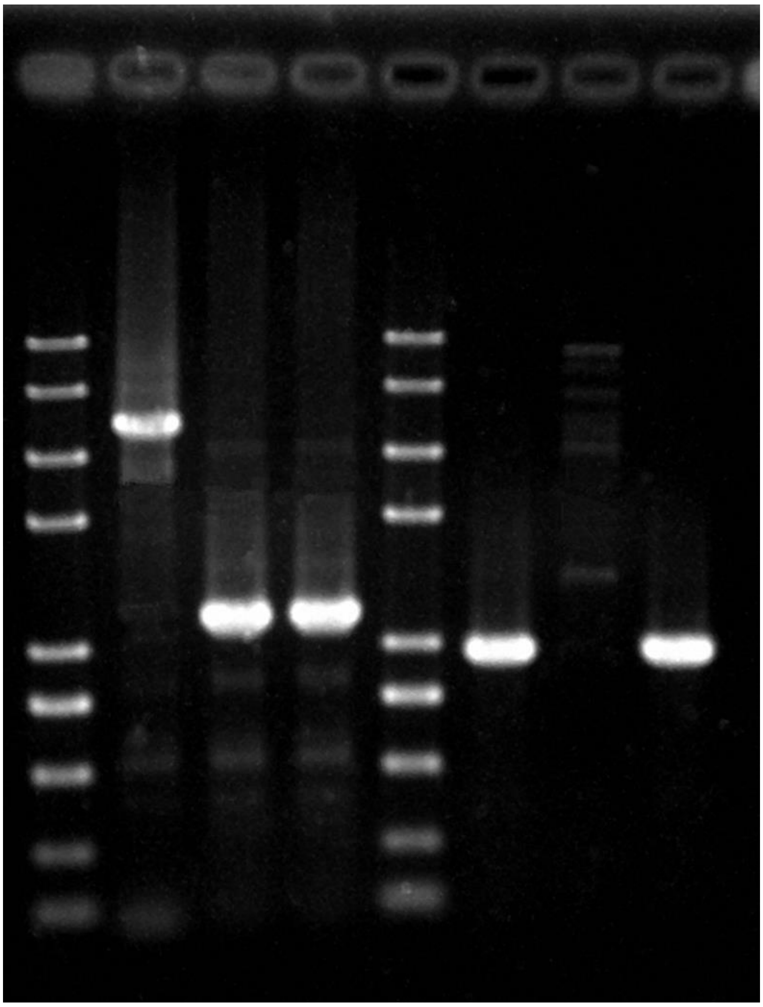

Figure S4. Full-length gel of diagnostic PCR confirming the mutant strains GW4-Δ*mcp* and complementation strain GW4-Δ*mcp*-C (**Figure S1 B-C**). Lane 3 and 4 were both *mcp* mutant strain, only lane 3 was presented. Amplicon identities were confirmed by DNA sequencing.

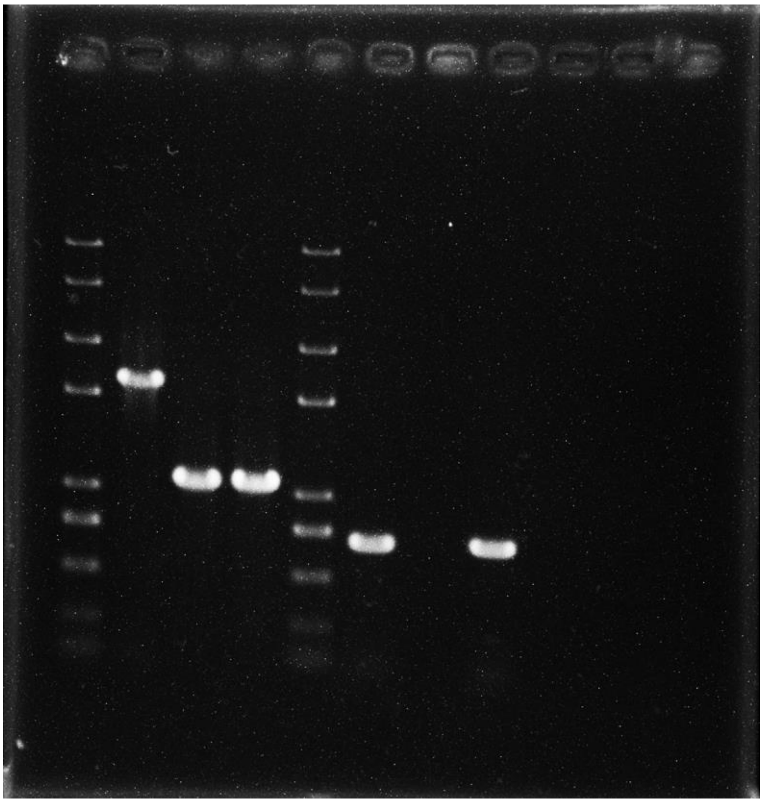

Figure S5. Full-length gel of diagnostic PCR confirming the mutant strains GW4-Δ*aioR* and complementation strain GW4-Δ*aioR*-C (**Figure S1 D-E**). Lane 3 and 4 were both *aioR* mutant strain, only lane 3 was presented. Amplicon identities were confirmed by DNA sequencing

Table S1. The strains and plasmids used in this research.

Strain/plasmid	Relevant properties or derivation	Source or reference
**Strains**		
Agrobacterium tumefaciens		
GW4	Wild type, As(III) oxidizing strain	Reference 6
GW4-Δ*aioR*	*aioR* gene deleted	This study
GW4-Δ*aioR*-C	Complementation of GW4-Δ*aioR*	This study
GW4-Δ*mcp*	*mcp* gene deleted	This study
GW4-Δ*mcp*-C	Complementation of GW4-Δ*mcp*	This study
GW4-Δ*aioA*	*aioA* gene deleted	Reference 15
Escherichia coli		
DH5α	*supE*44 *lacU*169(*φ80lacZM15*) *hRDR*17 *recA*1 *endA*1 *gyrA96 thi-1 relA*1	Reference 37
S17-1 λ*pir*	F^{-} RP4-2-Tc::Mu *aphA*::Tn7 *recA* λ*pir* lysogen; Sm^{R} Tp^{R}	This study
BL21Star^{TM} (DE3) pLysS	F^{-} *ompT hsdSB* (r_B^{-} m_B^{-}) *gal dcm me*131 (DE3) pLysS (Cm^{R})	Invitrogen
XL1-Blue	Δ(mcrA)183 Δ(mcrCB-hsdSMR-mrr)173 endA1 supE44 thi-1 recA1 gyrA96 relA1lac [F' proAB lacIqZΔM15 Tn5 Km^{r}]	Stratagene
**Plasmids**		
pJQ200SK	*sacB* *sac*^{R} Suc^{S}; Gen^{R}	Reference 38
pJQ-*aioR*	Gene mutation plasmid to create GW4-Δ*aioR*; *sacB* *sac*^{R} Suc^{S}; Gen^{R}	This study
pJQ-*mcp*	Gene mutation plasmid to create GW4-Δ*mcp*; *sacB* *sac*^{R} Suc^{S}; Gen^{R}	This study
pCT-Zori	oriT broad host range; Cm^{R}	Lab stock
pCT-Zori-*aioR*	*aioR* gene cloned into pCT-Zori; Cm^{R}	This study
pCT-Zori-*mcp*	*mcp* gene cloned into pCT-Zori; Cm^{R}	This study
pET-28(a+)	Km^{R}, His6 Tag expression vector	Novagen
pET-28a-*aioR*	*aioR* in frame fusion to the multiple sites of pET-28(a+)	This study
pET-28a-*mcp*	*mcp* in frame fusion to the multiple sites of pET-28(a+)	This study
pLSP-KT2lacZ	Km^{R}, *ori*V, *lacZ*-fusion vector uesd for *lacZ* fusion constructs	Invitrogen
pLSP-*aioBA*	pLSP-KT2lacZ containing *aioBA* promoter	This study
pLSP-*mcp*	pLSP-KT2lacZ containing *mcp* promoter	This study
pTRG	Tet^{R}, for bacterial one-hybrid assay	Stratagene
pTRG-AioR	pTRG containing AioR coding region	This study
pBXcmT	Cm^{R}, for bacterial one-hybrid assay	Reference 23
pBX-P*mcp*	pBXcmT containing *mcp* promoter	This study

Table S2. Primers used in this research.

Primer pair	Primer sequence	Use
AioR-up-F/<br>AioR-up-R	5'AAAGGATCCAACACCAGCCTGTCCCTTCT3'/<br>5'**GAGCTCGCGCATGGCCTGCTC**TCCCTTCGT<br>CCACCATTTGA3'	Crossover PCR to create *aioR* mutant
AioR-dn-F/<br>AioR-dn-R	5'**GAGCAGGCCATGCGCGAGCTC**GCATTTCA<br>CGCACGACACTC3'/<br>5'AAATCTAGAAACCTGCTGGCCTCCCTTTT3'	Crossover PCR to create *aioR* mutant
Mcp-up-F/<br>Mcp-up-R	5'AAAGGGCCCCAGAACAGCCGCTTGAGG3'/<br>5'**TTCGAAAATGGAATGAGC**GGCTGATTTGCG<br>TAGTTC3'	Crossover PCR to create *mcp* mutant
Mcp-dn-F/<br>Mcp-dn-R	5'**GCTCATTCCATTTTCGAA**GAATGAGGCGTG<br>GGAAGA3'/<br>5'AAAACTAGTGGTTACCTTGGGCTATGC3'	Crossover PCR to create *mcp* mutant
AioR-C-F/<br>AioR-C-R	5'AAAGTCGACCGTTCAGAGTGGTGATG3'/<br>5'AAAAAGCTTTCGCCGCCGTTGTT3'	Complementation for *aioR* mutant
Mcp-C-F/<br>Mcp-C-R	5'AAAGAGCTCGCATACGGAAAGCACA3'/<br>5'AAATCTAGATCCGCTTTCTGCTTCT3'	Complementation for *mcp* mutant
AioR-in-F/<br>AioR-in-R	5'CCCTTCATCGCCGTAAACTG3'/<br>5'CTGCTGCCTGGGAAATCTGT3'	Confirm for GW4-Δ*aioR* and GW4-Δ*aioR*-C
Mcp-in-F/<br>Mcp-in-R	5'AAGCGGGACTGTCTATGT3'/<br>5'AATGTGGCAAGCGTCT3'	Confirm for GW4-Δ*mcp* and GW4-Δ*mcp*-C
AioR-HTH-F/<br>AioR-HTH-R	5'AAAGAATTCGAGGAGATCATGCAGATCAGGG<br>3'/5'AAAGCGGCCGCAGTTGCCCGTTCGCTTTT3'	Over-expression of AioR
Mcp-As-F/<br>Mcp-As-R	5'AAAGAATTCATGTATTGGCAAGAAGTTGGA3'/<br>5'AAAAAGCTTTTACCGATGCATTGGTCCGAG3'	Over-expression of Mcp
AioR-lacZ-F/<br>AioR-lacZ-R	5'AAAGAATTCGCATTTCACGCACGAC3'/<br>5'AAAGGATCCGCCAGAAACGGCGACA3'	Cloning of *aioBA* promoter for reporter gene
Mcp-lacZ-F/<br>Mcp-lacZ-R	5'AAAGAATTCACGCATACGGAAAGCACA3'/<br>5'AAAGGATCCGGCTGATTTGCGTAGTTC3'	Cloning of *mcp* promoter for reporter gene
16S-RT-F/<br>16S-RT-R	5'GGTATGGGCATTGGAGACGA3'/<br>5'GGCAACTAAGGGCGAGGG3'	qRT-PCR for *16S*
CheA-RT-F/<br>CheA-RT-R	5'ATGCGTCGCTTTATTCG3'/<br>5'GCTGCGGATACCCTCTT3'	qRT-PCR for *cheA*

Table S2. Primers used in this research.

(Continued)

CheY2-RT-F/<br>CheY2-RT-R	5'CCCATCATCTCGTCATTTC3'/<br>5'TTCTTGGTGGTCGGGTT3'	qRT-PCR for *cheY2*
FliG-RT-F/<br>FliG-RT-R	5'AGGAACGGGTCTGATGG3'/<br>5'CGATGGTCTGCGGATG3'	qRT-PCR for *fliG*
AioR-one-F/<br>AioR-one-R	5'AAAGCGGCCGCAATGCAGCGTGAAACAGGA<br>3'/ 5'AAAGAATTCCCGCCGTTGTTCATTTGG3'	Cloning of *aioR* for bacterial one-hybrid
Mcp-one-F/<br>Mcp-one-R	5'AAATCTAGAGCGATGCCTGGAAAGAAA3'/<br>5'CAAATGCTGGCTCCGATT3'	Cloning of *mcp* promoter for bacterial one-hybrid
AioBA-Box-F/<br>AioBA-Box-R	5'TTTCACGCACGACACTCT3'/<br>5'ATTGCTATGCCAATTCCTA3'	Cloning of *aioBA* promoter for EMSA
Mcp-Box-F/<br>Mcp-Box-R	5'TACGGAAAGCACAATACG3'/<br>5'CTGGGATCGCATAGTTAA3'	Cloning of *mcp* promoter for EMSA
Mcp-M-F/<br>Mcp-M-R	5'TTGCAGTTGTCGGATTTCCGCTTATCATTG3'/<br>5'CGGAAATCCGACAACTGCAAACTGGTTTCA3'	Create mutant *mcp* probe

* The bolded sequences denote the reverse complement sequences for the crossover PCR, while the underlined sequence denotes the restriction enzyme sites.

References

37. Hanahan, D. Studies on transformation of *Escherichia coli* with plasmids. *J. Mol. Biol.* **166**, 557-80 (1983).

38. Quandt, J. & Hynes, M. F. Versatile suicide vectors which allow direct selection for gene replacement in gram-negative bacteria. *Gene.* **127**, 15-21 (1993).
